# The patch as method: The arts’ contribution towards understandings of conflict

**DOI:** 10.1177/01925121231177442

**Published:** 2023-06-02

**Authors:** Daniele Rugo

**Affiliations:** Brunel University London, UK

**Keywords:** Armed conflict, art, filmmaking, Lebanon, Didi-Huberman

## Abstract

This article asks: how do art practice and research give form to changing dynamics of conflict? Its argument is two-fold: art’s contribution can be developed from empirical considerations (what art finds out), and from methodological ones (how art finds something out). Bringing in art practice and the research methods it informs into political science helps understand conflict and its changes: by engaging simultaneously with the interaction between the collective and the personal, art practice and research elucidates those complex and layered narratives used by various actors in conflict that often resist approaches rooted in social and political sciences. By paying attention to everyday interactions and emphasizing dynamism, art provides a different way to chart changes in armed conflict. Art documents discourses that are difficult to communicate otherwise and allows us to detect and engage with the grey areas, transformations, processes and ambivalences of conflicts that escape neat categorizations.

The comportment of artworks reflects the violence and

domination of empirical reality by more than analogy.

(Adorno, 1997: 139)

War and armed conflict more generally play a foundational role in the history of western (but not only western) art. Homer’s *Iliad*, one of the earliest examples of western literary and visual culture, is an epic tale of war and warriors, and forms the blueprint for the historiography of a period for which we have limited records. This link should be attended to with more care and more imaginative power than we have thus far. Art might turn out to provide not just a second-hand rendering of experiences of war, but unique insights on conflict and its changes.

In dialogue with the other contributions to this Special Issue, this article seeks to contribute to ongoing debates on the insights the arts can offer to understandings of conflict and its changes. The argument does not attempt to treat the topic systematically or to exhaust all possible avenues, but to review existing features of the debate on art and conflict and develop some trajectories for further work. The research question this article pursues is: how do art practice and research give form to – and perhaps disrupt? – changing dynamics and experiences of conflict?’ In line with this Special Issue, this article proposes to ‘challenge – or relax the rigidness of – externally imposed categories and labels related to “armed conflict” ’ (Idler, 2023).

The argument offered here is not strictly speaking written from within the disciplines that normally deal with conflict, especially political science (see e.g. Dursun-Özkanca, 2023; Idler and Tkacova, 2023 ), economics (see e.g. Nogales and Oldiges, 2023), history and sociology or psychology (see e.g. Alderdice, 2023). It is developed from within aesthetic theory and draws on the author’s own practice as a filmmaker working on conflict.^1^ Consequently, rather than the more usual gesture of a political scientist asking what the arts bring to the epistemological make-up of her discipline, this article argues for what the art can offer to political science and related disciplines and where the specificity of such contribution might reside. Whilst the conclusions might not differ greatly, this argument is driven by a different sensitivity, a different set of concerns, and operates through a different critical lexicon.

The trajectories sketched here move from the idea that the arts can be decisive^2^ to the task of understanding conflict on an empirical level (*what they find out*) and on a methodological level (*how they find something out*). As Negash (2004) writes, ‘few steps have been taken to go beyond an appreciation of [art’s] mysterious potency to elucidate what in its characteristics helps us better understand and shape political ideas and practices’ (188).

Accounting for the dynamic character of armed conflict, emphasized by all articles in this Special Issue, the arts can play this role, however, only inasmuch as we first dislodge the question of art and conflict from the limits that are too often assigned to it. This article contends that the prevailing understanding of the arts as (merely) mimetic/representational tools, caught in a paradigm of originality versus simulation, hinders their contribution to understandings of how conflicts change. This article offers two reasons to look at conflict research from the arts (both practice and theory):

1. Art does not – or not merely – represent war, but presents it, makes it visible and audible, gives its experience a form; what goes on in art’s relation to conflict is a world-making process.

2. The reason why one would want artists (filmmakers, painters, poets . . .) to think about conflict is not to reinforce or ‘decorate’ what we already know, but because artists can produce disruptive thinking, giving form to that which might escape the threshold of ‘visibility’ or ‘tangibility’ and produce, therefore, a different knowledge of conflict and its changes.

Finally, every art form can serve war efforts and warmongering (Mitchell, 2012; Winter, 1992) as well help us to find ways out of conflict (Mitchell et al., 2020) and to imagine peace (Möller, 2019; Rugo and Weaver, 2021; Wenders and Zournazi, 2013). Similarly, art’s ability to stir emotional responses has been used both to consolidate consensus or cement the status quo and to disrupt and antagonize dominant power structures. The arts’ role becomes even more important given that, as Alderdice (2023) mentions in this Special Issue, recent conflicts show that the overwhelming physical force does not always bring victory. Whilst the article acknowledges art’s double function, the argument focuses on the arts as a disruptive force with the potential to reconfigure how we think about conflict and its changes across time and space.

Before we move to the core argument, it is helpful to define a set of terms that are central to this article. When discussing conflict, for instance, the argument adopts the same framework developed for the introduction to this Special Issue. Conflict is thus understood as ‘organized intergroup violence’, but also as a ‘dynamic social phenomenon’ (Idler, 2023) with a strong focus on ‘transnationality’. As a dynamic phenomenon, the article contends that analyses of conflict need to take into account both ‘perceptions’ and ‘experiences’. As a consequence, the use of terms such as ‘art practice’ and ‘the arts’ or more specific ones such as ‘filmmaking’ points to the hold these practices have on perceptual and experiential dimensions of conflict. Whilst of course creative practices – and filmmaking among them – have institutional and industrial frameworks that they respond to and emerge from, the use of these terms in this article emphasizes their ability to produce outputs that are experientially rich and capable of embedding changing perceptions of conflict. This means, for instance, that the article does not treat filmmaking as an industry, but emphasizes this practice’s ability to contribute – as an art form – to producing insights about changes in conflicts and communicating the perceptions of those who live through them (Möller and Shim, 2019; Townsend and Niraula, 2016). The term ‘representation’ – connected to the argument made here about art practice – is one that also requires a short definition. Art is normally understood to produce representations, objects that are ontologically different from the phenomena they picture (Bolt, 2004). Following this paradigm, one refers to images as existing in a realm separate from our everyday reality. This article offers a non-representational approach, which can be defined as the attempt to move beyond the separation between image and reality, by thinking of images as having impacts on our everyday lives. Finally, this article introduces the term ‘patch’ – borrowed from art historian Georges Didi-Huberman (1989) – to describe how the opacity and excess of art practice vis-à-vis instrumental logic brings us closer to an understanding of conflict as a dynamic social phenomenon.

This final point also introduces the theoretical contribution of this article, which rests on the idea that art documents and articulates discourses and experiences that are difficult to communicate otherwise. In this way, the article aims to collaborate with mainstream political science work on conflict dynamics and long-term changes (Kaldor, 2006; Kriesberg and Neu, 2018; Strachan and Scheipers, 2011) by stressing the need to detect and engage with the grey areas and ambivalences of conflicts that escape neat categorizations.

## The turn and its limits

Since the goal here is to offer an alternative understanding of what the arts can do, I begin by addressing the specific epistemology of art in relation to conflict. By interrogating this epistemology in its specificity and uniqueness, one can understand, through the arts, something about conflict that other analytical lenses overlook or minimize. The approach therefore aims to identify the specific gesture of the arts in relation to conflict. It would be counter-productive to conduct one more argument based on the juxtaposition of the arts, their findings, insights and explicative power to that of other methods, rooted – say – in quantitative analysis and whose evidentiary power is far more documented and accepted. It is also not a matter of drawing comparisons, ranking disciplines in terms of how effective they are. To begin this exercise, one can recall Didi-Huberman’s argument on our treatment of images to point to a prevailing tendency. In his discussion of the four photographs taken by members of a Sonderkommando and snatched from the hell of Auschwitz-Birkenau in 1944, Didi-Huberman stresses how these images demand a sustained exercise of the imagination. One needs to imagine, even before and beyond analysis. Imagining would take a double meaning here. On the one hand, it is a matter of exercising the imagination, of attending to nuances, ambiguities, idiosyncrasies, of looking into patches, blind spots, areas of reduced visibility. In other texts – to which this argument will return later on – the philosopher insists on the work done by ‘patches’ and other ‘formless zones’ (Didi-Huberman, 1985: 44; 1989: 149). It is therefore a matter of taking the partiality of images as a trigger to reframe the understanding of conflict, rather than reducing images to mirrors of analytical positions formulated and organized elsewhere. On the other hand, it is a matter of thinking through images, thus with the specificity of the image, with what the image reveals in excess of its evidentiary power and of representational regimes. Didi-Hubermann (2003) expresses the bias that informs a reductionist view of images, by saying that we always ask both too little and too much of them (32–33). We want the whole truth and so complain because they neither hold nor communicate this truth. They are too partial, too much is left outside the frame, too many areas are poorly lit and too much unnecessary material obstructs our view of the main subjects. We also complain, however, about their status and – judging them according to a representational rule – consign them to the status of copy, therefore invalidating whatever insight they might provide as sheer seduction. Didi-Hubermann (2003) adds, though, that we might fail images even when we take them seriously as documents: ‘by immediately relegating them to the sphere of the document [. . .] we sever them from their phenomenology, from their specificity, and from their very substance’ (33). In both cases we fail to pay enough attention to them. Or rather we fail to pay the right kind of attention.

Whilst it is true that the arts remain marginal, in the last 15 years the discipline of international relations (IR) has undergone what Bleiker (2001) named an ‘aesthetic turn’. Even before Bleiker, Bokina (1991), in an issue of this journal dedicated to ‘The Politics of Art’, had already noted that

in comparison to research on hearty and palpable political subjects such as constitutions, government institutions, military strength, parties, interest groups, or public policy, explorations of the politics of a painting or a play appear ephemeral, peripheral, perhaps even capricious. This need not be the case. (3)

Similarly, Bleiker (2009) challenged the idea that the arts are too frivolous to take on subjects such as political violence and world politics more generally and insisted on the need for aesthetic inspiration to ‘find innovative solutions to entrenched conflicts and difficult political challenges’ (1). Pitting Deleuze’s divergent thought against Kant’s harmonious common sense (Bleiker, 2009: 29), Bleiker calls for a renewal of IR performed through the aesthetic, a renewal that would open a space for thinking and produce ‘a shift away from the harmonious common sense imposed by a few dominant faculties towards a model of thought that enables productive flows across a variety of discordant faculties’ (Bleiker, 2001: 519). The aesthetic turn has fuelled an increased attention for the visual, expressed often in terms that describe images’ ability to mediate political events and to be ‘significant objects that play a role in shaping our understanding of international affairs (Berents and Duncombe, 2020: 567). In a similar way, the aesthetic turn has also collaborated with a more generous engagement with emotions and affects (Hall, 2015), as tools that can challenge the perception of world politics ‘as a realm where, above all, precision, instrumentality and hence a technical, calculated, emotional-less rationality must necessarily prevail’ (Hutchinson, 2016: xi).

For Bleiker and those who developed similar arguments (Carruthers, 2001; Hozic, 2017; Kompridis, 2014; Moore and Shepherd, 2010; Shepherd, 2008; Sylvester, 2001), the aesthetic should be seen as the site from where to open new lines of enquiry around representational politics, gaps in representation and – in a move faithful to much of Foucault’s work – representation as a site of power.

The turn identified some important features of the aesthetic: its ability to challenge the logical integrity of other methods and expand as a consequence the type of discourses, subject matters and objects of enquiry; its ability to show the particularity of claims advanced and to show the power relations at the heart of our descriptive or analytical statements, but also its insistence on the fact that every object of study is encountered via a mediation and not in-itself. As Harman (2019) puts it, ‘film depicts the rhythms and temporality of the everyday and the hidden dimensions of politics that cannot be captured by the written word’ (15). Callahan (2020) broadens the scope considerably by working on the dyad visibility/visuality, where the former ‘deconstructs’, whilst the latter ‘creates’ (20). This overcoming of a purely hermeneutic position shows how visuals can ‘provoke social-ordering and world-ordering practices’ (Callahan, 2020: 32) that collaborate with the creation of ‘affective communities of sense’ (41). The fact that the arts foreground questions of rhythm and speed, but also that they are predicated on a movement of creation and undoing, makes them particularly apt at approaching conflict as a dynamic and layered phenomenon, rather than a static one.

As Moore and Shepherd (2010) argue, however, despite the effort of a relatively small number of scholars to put the aesthetic back into the study of world politics and conflict, ‘there has been relatively limited engagement between various bodies of literature that could be seen to constitute this turn’ (299). In other words, in relation to political violence, war and conflict, art is still a kind of ‘subjugated knowledge’ (Foucault, 1980: 82); both a knowledge that is repressed and that has the potential to be disruptive. Simply put, art is hardly ever invoked when it comes to studying conflict; it is considered a naive knowledge ‘located low down on the hierarchy, beneath the required level of cognition or scientificity’ (Foucault, 1980: 82). Bleiker (2009: 2) correctly identified aesthetic thinking as a fringe knowledge that challenges accepted opinions on political violence. Distance and objectivity, for instance, are meant to be foundational principles to study conflict. Nevertheless, creative practices show how the personal and the affective are crucial to understand violence, as is also pointed out by Alderdice (2023) in this Special Issue who – by drawing on insights from psychology – demonstrates the relevance of ‘devoted actors’ rather than ‘rational actors’ to understanding changing conflict dynamics. Art questions this unity, this systematizing effect where everything can be explained, and everything is centralized around one single way of thinking. Part of the argument in the present article is then dedicated to trigger some of this discontinuous, illegitimate, disqualified knowledge, to render it capable of opposition, of providing a different insight into conflict, to open ways of thinking, doing and seeing governed by the potential of the imagination. The first insight that the arts afford us is the insistence on conflict as experienced (see also Alderdice, 2023). Thinking about conflict as experienced means bringing to the front elements of war that are traditionally excluded, looking for deeper ways in which war reaches into society (for instance, emotional, affective and personal trajectories); it means understanding conflict not as a discrete, static event, but as a dynamic continuum, therefore starting earlier and going on for longer. It can even unhinge our understanding of the category ‘armed conflict’ as such. It also means abandoning the idea that we can always fashion a coherent narrative, but that we have to acknowledge the complex, dynamic, and pervasive (everyday) character of conflict. Just as Idler and Tkacova (2023) visualize in this Special Issue, the shifting constellations of multiple actors involved in conflict – with new dominant actors emerging and ‘old’ ones disappearing or re-emerging – undermine dyadic explanations of conflict. In a very similar fashion, the arts operate from the start according to a non-linear, multi-faceted and dynamic model of conflict. This approach also suggests that we have to commit ourselves to a conflict’s least tangible but nonetheless life-changing impacts. As feminist scholar Laura Sjoberg (2013) writes:

[S]tarting with the lives of people, gives us not just a different method of studying war, but a different view of war, one which draws our attention away from national interest politics to the individuals that touches and is touched by war physically and emotionally. (253)

Art can therefore be disruptive, not despite the fact that it is partial, subjective, tuned into emotions, but precisely because of this, because it gives form to perpetrators and victims’ rationalities, perceptions, motives and experiences, which challenge the ways in which we understand political violence.

Considering experience as a key element of what the arts offer allows one to express a certain dissatisfaction with the aesthetic turn and its excessive emphasis on the arts as representational tools, which (explicitly or implicitly) sees the turn subscribe to the representational regime and its aporias. In doing so, the aesthetic turn has somehow undone its very premise – that of using aesthetic thinking and aesthetic experience to explode the field. One way to return to that promise, or at least to continue some of the work, would be to insist on the work of the arts as taking place outside the representational regime. In speaking of the arts as defined by specific regimes, this article follows Rancière’s (2004) elaboration in his seminal *The Politics of Aesthetics*. For Rancière, the representational regime – or regime of representational mediation (2010: 138) – is one dominated by *mimesis*, which organizes ‘ways of doing, making, seeing, and judging’ (2004: 13).

This means moving beyond the idea that the arts offer one more identification with the world, one more way to bridge the gap between reason and its object, subjects and their experiences. Perhaps the turn could have insisted more on art as that which, in presenting a world, renegotiates the world anew, and therefore presents the world as strange. In his *Aesthetic Theory*, Adorno captures this movement away from representation in a way that, whilst cryptic, is nonetheless helpful to a discussion of conflict, by saying that an image ‘is not a copy of the event, but the cypher of its potential’ (1997: 32).

## Representation and dissemblance

To offer a more detailed account of how the arts, released from the confines of the representational regime, can support our understanding of conflict dynamics, the article turns to the question of ‘war representations’. This analysis will offer an alternative, based around the idea not of identification mentioned above, but of dissemblance. Dissemblance, rather than verisimilitude, becomes the very engine of a possible intersection between the arts and armed conflict. The idea – aptly expressed by Bolt (2004) – is that art produces a ‘dynamic material exchange’ (8).

Fredric Jameson advances a structured critique of the relation between the arts (in particular literature and film) and war. It is worth quoting a passage at length:

[O]ne often has the feeling that all war novels (and war films) are pretty much the same and have few enough surprises for us, even though their situations may vary. In practice, we can enumerate some seven or eight situations, which more or less exhaust the genre. If so, and despite experience that confirms this opinion, this would be an astonishing fact, given the radical changes in warfare historians document since the hand-to-hand combat in the plains before Troy. (Jameson, 2009: 1533)

Jameson concludes that this aesthetic conformism is due to what he calls the unrepresentable nature of war. He surveys an impressive array of – mostly literary – examples, from Stendahl to Tolstoy, from the *Simplicissimus* to Doblin and Kluge. Despite their immense value to literary culture, these works fail to accomplish their task: representing war *fully*. They can rather be situated at one of two poles: abstraction and experience. These poles do not come into contact with each other, but are caught in a dialectic that seems to exclude in turn one or the other and thus spoils the wholeness of any representation. This wholeness that Homer had is now lost and irretrievable. Since wholeness is lost forever, a totality of representation or a represented totality as its epitome is also irretrievable. Armed conflict is a collective reality for which we have no representation. Jameson (2009) therefore concludes: ‘War is one among such collective realities, which exceed representation fully as much as they do conceptualization and yet which ceaselessly tempt and exasperate narrative ambitions, conventional and experimental alike: unless, of course, this particular reality ceases to exist’ (1547). One could look at the problem from a different point of view. The issue perhaps is not so much with war as unrepresentable, but with the idea of representation itself and in particular with the idea that one could conceive of a full representation. If war is not a candidate for a whole representation, what sphere of experience would totally fit within its corresponding representation? Why also should one insist so much on the idea of art as another type of representational knowledge? Why do we want art to be another place where we recognize the world and our experiences? Once art is taken as another site for recognition, it is in effect inaccurate and vague, but also overwhelming in the wrong sense. As Deleuze (2005) writes in relation to the work of the British painter Francis Bacon, representation ‘implies the relationship of an image to an object that it is supposed to illustrate’ (2–3). Deleuze (2005: 3) therefore suggests looking for a non-illustrative relation, one that acts ‘beneath or beyond representation’ (46). From this, one could move to the thought that this inability to ‘represent’ could be less problematic if we were less obsessed with the idea that all the arts do is represent. Once we move away from this representational idea, we might see different ways in which art contributes to an understanding of conflict. By taking art only as representation, we assume it to be an activity that reproduces the form of something it is nonetheless external to. This conception implies that artworks always illustrate an original and are judged on the basis of how accurate an illustration they provide. In this sense, they always fall short. This argument contends that unless one moves away from a representational understanding of art, it will be difficult to gain anything from the arts in relation to conflict. On the other hand, once we do move away from this restrictive regime – and take artworks themselves to be akin to ‘original experiences’ – we might be able to open up new ways of looking at the intersection between conflict and the arts. The key here is to understand artworks as part of the reality they present. When art gives form to a conflict, it shapes it and contributes to its articulation. We should look at the arts not because they have a descriptive power, but precisely in what is non-descriptive about them and not simply because they lead to an epistemic rethinking of the foundations of studies of conflict, but because the very non-descriptive, sensuous, non-identical elements of arts find an echo in experiences of conflict themselves. They find echoes and resonances in those residues of experience that are not easily expressed, precisely because their density resists being channelled into an intelligible whole. Something of this density and tension of the arts is proper to the experience of changing conflict dynamics themselves.

The arts work not according to the similar, but to the dissimilar. They turn away from the thing, precisely to bring it into focus. For Adorno, art presents non-identity, opening the possibility of something to shine beyond our ability to represent it (to make it identical to the epistemological categories we have). A considerable part of the aesthetic turn is thinking about representational politics again in terms of how the representation is adequate either to the original event or to a certain political agenda that the image maker or user wants to push. Perhaps the aesthetic turn should have insisted more on the stain, the patch, the blotch, the catastrophe of identitarian thinking that the arts bring about. Insisting on the representational regime (even to highlight how sites of representations matter) implies an embracing of identitarian thinking, which the arts resist. As Rancière (2008) writes, ‘what art produces is not rhetorical persuasion about what must be done [. . .] it is the multiplication of connections and disconnections that reframe the relation between bodies [. . .] It is a multiplicity of folds and gaps in the fabric of common experience that change the cartography of the perceptible, the thinkable and the feasible (72). Therefore, the affect proper to the aesthetic is one of ‘dis-identification’ (Rancière, 2008: 72). Rancière motivates this not just as the effect of art, but as its very operation. The gap between outcome and intention is one that can find no reconciliation (and bars, therefore, the efficacy of the representational): ‘no art can avoid the cut that separates outcomes from intentions and precludes any direct path towards another side of words and images’ (Rancière, 2008: 82). In other words, art can capture what reflective logical thinking cannot. (In this Special Issue, Alderdice (2023) pursues, albeit deploying a different language, a similar argument about the significance of non-rational functioning to an understanding of conflict.)

Where can one find readings that support this argument? One could start by interrogating the way in which the arts ‘perform’ conflict, offer an insight into the performance of violence and what it means to live with it. What would be crucial here is to understand this ‘performance’ element, which is very different from a ‘representation’. The artwork in conflict creates and puts us in contact with the condition and experience of violence. Further support for our reading could come from those theorists of art for whom art is not a separate sphere of human activity, entertained with a degree of detachment, but rather an immersion into the human condition, one that is done within the space of everyday life. The aesthetic is not a sphere that exists on the side of human experience, it is part and parcel of that experience. The American philosopher John Dewey (1934) writes in the opening pages of *Art as Experience*:

When artistic objects are separated from both conditions of origin and operation in experience, a wall is built around them that renders almost opaque their general significance [. . .] Art is remitted to a separate realm, where it is cut off from that association with the materials and aims of every other form of human effort. (3)

From this point of view, *The Creative Memory of the Syrian Revolution*^3^ stands as a striking instance of this. The database uses art produced during the Syrian uprising and the subsequent conflict to show not how the conflict is represented, but how art is part of the very experience of that conflict. Using the website means experiencing the conflict from an arts-based approach. It is a chronicle of the war through art produced from within that conflict. Importantly, this project also says something about how the nature, production and distribution of creative/artistic artefacts have changed over the last 20 years and keep changing. Whilst it is arguable whether access to the means of creative production has substantially increased, it is fair to say that digital media has made this production far easier to archive and disseminate. Two partial observations can be made: these works exist now not in the separation we have come to expect of artworks. They are rather part of an ecology marked by abundance and accessibility, but also one that pushes works to remain open to processes of remixing and recontextualizing. The second is that the fact that these works avoid the separation between art and life evoked by Dewey and are often produced by citizens-artists inevitably makes their status ambiguous. They often inhabit the threshold between factual reporting and creative production, and it is their blurring of these categories that gives them force.

Those using the arts in peacebuilding and conflict prevention activities have already moved away from the representational model. The arts here are used for a variety of reasons, none of which seems to insist on their representational power. They provide soft engagement; they are good entry points for those groups that are difficult to engage and are good for initiating encounters between opposing parties. They offer a space where groups can come together and do collective work. These tools offer individuals the ability to narrativize and communicate their experiences, thus breaking through the barrier that often cuts people in conflict off from each other and the rest of the world (whether combatants and victims). In 2019, for instance, we held a series of screenings of our film *About a War* (2018) across Lebanon (see note 1). The screenings were either targeted at a specific audience or open to the general public, and the Q&As featured one or more of the former militia fighters who are the protagonists of the film. These screenings triggered particularly important conversations in contexts where the audience was composed of young people who had themselves taken part in more recent conflicts in Lebanon or Syria: young people related to the film, not because of the accuracy of the filmic representations, but because the film expressed the older fighters’ experience in ways that mirrored their own. In this case, the film was the very site where that experience occurred, not merely a simulacrum of the experience. In addition to this, the scope of these conversations and what triggered them went beyond what we (as filmmakers) could anticipate or had planned. Often the trigger was not a coherent testimony or a significant detail, but something marginal, maybe not clearly defined, a patch. This screening produces a first answer to the question that guides this article: how do art practice and research give form to changing dynamics and experiences of conflict? Art practices articulate experiences that cross generational divides and communicate these experiences by insisting on those grey, indefinite and marginal areas that are overlooked by most analytical frameworks.

## Towards an epistemology of the ‘patch’

If the arts are already used in terms that decentre the idea of ‘representation’, one can move a step closer to the specific epistemology of the arts in relation to changing conflict dynamics. Humans use the arts to document and chronicle conflicts they are involved in, to make sense of and understand conflicts (to explain them to themselves), to memorialize them, to protect themselves from the traumas caused, later on to heal this trauma, to get over them. Art can channel the affective and the experiential elements of humans’ relation of conflict, elements that whilst extraordinarily important for those who live conflicts on an everyday basis tend to remain opaque and largely excluded from other approaches.

Art articulates a discourse that cannot be articulated otherwise, neither in the political sphere, nor in the analytical one. This is the case for two connected reasons: art engages with a dimension that is either barely phenomenological (invisible, inaudible) or too dense to be easily condensed into analysis. There is a richness to art’s relation to conflict that cannot be dispelled or translated into either the economy of politics or that of analysis. This richness is what in the arts is most useful for our understanding of conflict.

Any approach that focuses on human-security (see also Nogales and Oldiges, 2023) cannot bypass the affective element. Conflict analyses must find a way to tap into emotions, since violence often has considerable emotional impacts (even if we do not want to talk about the emotional causes of it). Storytelling, for instance, speaks at a level that is otherwise ineffable, speaking of the incommunicable things that people in conflict experience. This of course also implies a series of drawbacks, the main one being that we are never fully able to ‘use’ the arts. As Hawksley and Mitchell write, ‘the arts are not just ambivalent, but volatile. When we deliberately utilize the arts for one extraneous purpose or another, whether we intend it for good or ill, we find that their power does not lie entirely within our control’ (2020: 11). This impossibility of using the arts in ways that can completely anticipate an outcome – to which this article returns in the conclusion – is also what in the arts is most deserving of attention. Rancière calls this effect ‘dis-identification’. This produces a sensuous rapture in our experience of the everyday, a feeling of strangeness. As Rancière (2008) writes, ‘on the one hand the effect escapes the strategy of the artist; on the other, the artistic strategy completes the process of dis-identification’ (73). In other words, the arts produce an effect that evades measure and calculation, but its force and significance rest precisely on this.

If it is true, as Kaldor (1999) argued, that previously accepted dichotomies of conflict do not work anymore, then art is perfectly placed precisely because it always works in multipolar ways. More importantly, if the analysis of conflict is to move away from rigid patterns, then art, which functions through non-linear time and fractured spaces and whose work is tensional (operating through tensions rather than essences), can lead us to uncover areas we find difficult to identify, navigate and communicate. Conflicts are full of grey areas and as long as we simply try to make them fit into neat analytical boxes, we will simply miss them; see, for example, in this Special Issue, Dursun-Özkanca’s (2023) analysis of Cyprus–Turkey’s frozen, unresolved conflict. Art can point us in the right direction; for instance, in understanding the changes in the wider impacts and long-term consequences of conflict, beyond the count of battle deaths, across state borders (see Idler and Tkacova, 2023), but also by addressing non-somatic forms of violence.

This means also accounting for those experiences that are marginalized and excluded not just by the geopolitics of knowledge production (a determining factor), but by the very onto-epistemology that still seems to dominate accounts of conflict. The question is similar to the one posed in different ways by Judith Butler and Achille Mbembe. Butler asks why certain lives are grievable, whilst others are not. Her response is that this has to do with the value imparted to certain lives by our frames of understanding. She writes: ‘Efforts to control the visual and narrative dimensions of war delimit public discourse by establishing and disposing the sensuous parameters of reality itself’ (Butler, 2009: xi). Within these parameters are decisions as to who is allowed to live, which body counts as grievable and who is abandoned to death. To this effect, Butler writes:

‘[T]his will be a life that will have been lived’ is the presupposition of a grievable life, which means that this will be a life that can be regarded as a life, and be sustained by that regard. Without grievability, there is no life, or, rather, there is something living that is other than life. (2009: 15)

In discussing colonial violence, Mbembe analyses the link between the conceptual development of democracy and the parallel development of colonial rationality. Colonial sovereignty creates a crowd of people whose life is ungrievable: ‘this life is a superfluous one, therefore, whose price is so meager that it has no equivalence [. . .] As a rule, such death is something to which nobody feels any obligation to respond’ (Mbembe, 2019: 38). What is put to death is a thing; the violence that meets it is violence against a thing, an ‘empty figure’ (Mbembe, 2001: 173). For both Butler and Mbembe, the value of life appears only under conditions, conditions in which its loss would matter (see Nogales and Oldiges’ (2023) article in this Special Issue on how deprivation intersects with the changing nature of conflict). So why is art helpful to address the fact that certain lives are under erasure, crossed out as lives? In Adorno’s understanding, art emphasizes the fact that our dominant ways of thinking privilege the identification of selected traits that fulfil – rather than interrupt – the perpetuation of meaning. That which interrupts and creates a gap, a break in the way our knowledge is produced and in what our knowledge produces, is resisted. Material is reduced to a function, but if that material does not fit the function, it is sidelined. As Rajaram writes, the arts can help in ‘breaking the epistemological imperative to find overarching structures as well as providing a moral grounding in the irreducible value of individual experience. This is then perhaps a way of beginning critique with the marginalized experience of the other: an ongoing processual critique of the fixity of identities that simultaneously resists the marginalization of lived identity’ (Rajaram, 2002: 370).

## Conclusion

It is here that a lesson can be learned from the ‘patches’ and ‘blotches’ that Didi-Huberman discusses in relation to painting. In his discussion of Vermeer’s work, Didi-Huberman juxtaposes the patch – a block of colour – with the detail as the element that can offer a different account of a work and dislodge our way of thinking. The patch, unlike the detail, neither describes nor allows for an iconographic reading. It disrupts depiction; it has an almost ‘explosive power; it is paint considered as a “precious” and traumatic material cause’ (Didi-Huberman, 1989: 149). The patch cannot be named; it attracts, but this attraction can neither be explained away, nor completely satisfied in recognition. As Adorno would say, it remains strange, it is suspended in strangeness and yet opens us up and opens the artwork to us. The detail, on the other hand, opens up the work to an explanation:

[W]hether thread, needle, knife, corkscrew, or navel, it partakes of the descriptive finesse which slices up and names the visible. The discovery of detail consists in seeing well something that is ‘hidden’, because minuscule, and in naming well what one sees. (Didi-Huberman, 1989: 165)

The detail is a piece of the paradigm, of the structure of a narrative that we know, but need to pay close attention to.

The patch is the disturbance in the paradigm that makes everything unstable. As Didi-Huberman writes, ‘the patch is therefore not just the sign-cum-clue of a dissimulated paradigm, a paradigm in absentia; more importantly, it is the sign-cum-clue of a labile, unstable paradigm. Therefore it is somehow twice removed from the order of rationality’ (1989: 164). The patch turns us away from the idea that everything can be seen and therefore recognized – that the only problem is that what we are looking for might be hidden or quite small, whilst our tools are the right ones:

[The] detail is defined; its contour delimits a represented object, something that is taking place or rather has its place in the mimetic space; its existence within the topos of the picture is thus specifiable and localizable, like an inclusion. The patch, on the other hand, rather than delimiting an object, produces a potentiality: something happens, passes, wanders around. (Didi-Huberman, 1989: 164)

The patch points to the need for a reconfiguration of our way of looking. The patch is hidden, but in a different way, and

[it] exists only as a result of not seeing well; it demands only to look, to look at something ‘hidden’, because it is obvious, there in front of us, dazzling, but difficult to name [. . .] the patch stares you in the face, mostly in the foreground of the picture, frontally, indiscreetly; but for all that it does not let itself be identified or enclosed: once uncovered it remains problematic. (Didi-Huberman, 1989: 165)

We cannot see the patch until it explodes in front of our eyes. Even in this explosion, we do not consume it through our explanation, we do not completely exhaust it; rather, it might reconfigure our thinking, away from the idea that we can consume and therefore exhaust and do away with a problem. It suggests the kind of processual understanding mentioned by Rajaram and embraced by philosophers such as Whitehead and Simondon.^4^

Maybe the problem when it comes to art’s ability to contribute to an understanding of conflict (including with the literature of the aesthetic turn) is that one cannot resist turning a patch into a detail, art into purpose. However, this is precisely the point where one encounters a resistance and either the aesthetic turns away or gets diluted into whatever function it can still be made to serve. The very possibility foreshadowed by Adorno, that art prefigures reconciliation, has to do with the fact that art shows itself resistant to being turned into a ‘purposive whole’ (Ranjaram, 2002: 354). As Adorno (1997) writes: ‘remote from reality, the purposiveness of artworks has something chimerical about it’ (139). The purposiveness of art is not something that can be turned quite simply into an agenda or a programme; something will always resist this transformation. Perhaps this lack of purposiveness needs to be acknowledged as such, and it is in this acknowledgement that art’s contribution to conflict finds its intensity and significance.

## Notes

1. See *About a War* (UK/Lebanon 2018, 82 min): https://www.iterationsfilm.com/about-a-war-doc.

2. I deliberately resist the idea of the arts’ *use* and therefore I am shying away from the term *usefulness*, because this would suggest an instrumentality that is part of the very limits I challenge.

3. See https://creativememory.org/en/archives/

4. For more on the nexus between violence, art, and process philosophy see Lübecker (2019).
